# Unbearable suffering while working as a nurse during the COVID-19 pandemic: A qualitative descriptive study

**DOI:** 10.1016/j.ijnsa.2023.100127

**Published:** 2023-04-12

**Authors:** Chloé Littzen-Brown, Hanne Dolan, Angie Norton, Claire Bethel, Jennifer May, Jessica Rainbow

**Affiliations:** a The University of Portland, School of Nursing and Health Innovations, Portland, Oregon, United States of America; b Arizona State University, Edson College of Nursing and Health Innovation, Tempe, Arizona, United States of America; c The University of Arizona, College of Nursing, Tucson, Arizona, United States of America; d University of Pittsburgh Medical - Community Osteopathic Hospital, Pittsburgh, Pennsylvania, United States of America; e Duke University, School of Nursing, Durham, North Carolina, United States of America

**Keywords:** Burnout, Covid-19 pandemic, Nursing workforce, Nurse well-being, Qualitative descriptive, Suffering

## Abstract

**Background:**

The COVID-19 pandemic resulted in negative consequences for nurse well-being, patient care delivery and outcomes, and organizational outcomes.

**Objective:**

The purpose of this study was to explore the experiences of nurses working during the COVID-19 Pandemic in the United States.

**Design:**

This study used a qualitative descriptive design.

**Setting(s):**

The setting for this study was a national sample of nurses working during the COVID-19 pandemic in the United States over a period of 18 months.

**Participants:**

Convenience and snowball sampling were used to recruit 81 nurses via social media and both national and state listservs.

**Methods:**

Using a single question prompt**,** voicemail and emails were used for nurses to share their experiences anonymously working as a nurse during the COVID-19 pandemic. Voicemails were transcribed and each transcript was analyzed using content analysis with both deductive and inductive coding.

**Results:**

The overarching theme identified was Unbearable Suffering. Three additional themes were identified: 1) Facilitators to Nursing Practice During the COVID-19 Pandemic, 2) Barriers to Nursing Practice During the COVID-19 pandemic, with the sub-themes of Barriers Within the Work Environment, Suboptimal Care Delivery, and Negative Consequences for the Nurses; and lastly, 3) the Transitionary Nature of the Pandemic..

**Conclusions:**

The primary finding of this study was that nurses experienced and witnessed unbearable suffering while working during the COVID-19 pandemic that was transitionary in nature. Future research should consider the long-term impacts of this unbearable suffering on nurses. Intervention research should be considered to support nurses who have worked during the COVID-19 pandemic, and mitigate the potential long-term effects.

**Tweetable abstract:**

A study on nurses experiences during the pandemic reveals their unbearable suffering. Read here about the reasons nurses are leaving.

**What is already known**

● The COVID-19 pandemic has resulted in negative patient, nurse, and organizational outcomes.

● Globally, nurses are leaving or planning to leave the workforce at unprecedented rates.

Alt-text: Unlabelled box

**What this paper adds**

● Descriptions from a large national sample of nurses about their experiences working across diverse settings over 18 months of the COVID-19 pandemic.

● Nurses were negatively impacted by unbearable suffering while working during the COVID-19 pandemic, some resulting in new-onset alcohol abuse and suicidality.

● Specific facilitators and barriers to working as a nurse during the COVID-19 pandemic were identified that are transferable to nurses across settings.

● The transitionary nature of the pandemic was demonstrated, revealing that although nurses had similar experiences, they were dependent on geographical location and time.

Alt-text: Unlabelled box

## 1 Introduction

The COVID-19 pandemic brought the global healthcare system, including nurses, and other health and social service providers to a breaking point. This has led to negative consequences for nurse health and well-being, patient outcomes, and retention (Fernandez et al., 2020; Galanis et al., 2021;Joo and Liu, 2021;Tolksdorf et al., 2022;Zipf et al., 2021). A meta-analysis on the mental health among nurses (total sample size of 44,165) during the first 11 months of the pandemic found the prevalence of depression to be 22% and anxiety 29%, respectively (Ślusarska et al., 2022). Patients experienced delays in care (e.g., >4700 deaths per year in England are attributable to 3-month delays in cancer surgeries), and care rationing impacted outcomes (Sud et al., 2020). Globally, nurses are leaving, or planning to leave, at considerable rates – in the United States (US), the American Association of Critical-Care Nurses found that 66% of respondents (*n* = 6000) feel their experiences during the pandemic have caused them to consider leaving nursing altogether (AACN, 2021). Overall, the COVID-19 pandemic has left a mark on nurses and created questions about the future of our discipline. An understanding of nurses’ experiences working during the pandemic across settings and time is needed to develop generalizable interventions to support their health and well-being – ultimately improving patient and organizational outcomes. Thus, the purpose of this study was to explore the experiences of nurses working in healthcare settings during the COVID-19 pandemic through qualitative data collection using voice mail technology over an extended period of 18 months.

### 1.1 Theoretical framework

The theoretical underpinnings for this study was that of Guerrilla Theorizing, also referred to as “Theories in the Wild”, where nursing knowledge is developed in practice, within the person-environment processes of change. Guerilla theorizing is the unconventional, culturally sensitive, and innovative approach to knowledge development. In guerrilla theorizing, the voices of patients and their bedside caregivers are represented, and knowledge is developed *with* and not only *for* patients, their families, and nurses (Reed, 2018). The COVID-19 pandemic emerged abruptly, capturing the current experiences of nurses working during this important point in time was imperative. Thus, the design and methodology for this study was innovatively developed with sensitivity to nurses and healthcare systems being challenged and stretched to the limits.

## 2 Methods

A qualitative descriptive design was used to provide a rich description of nurses’ experiences working during the COVID-19 pandemic. A qualitative descriptive design is best used when there is little known about a phenomenon, ultimately providing understanding (e.g., what, who, and how) of participants' first-hand experiences. The findings of a qualitative descriptive design stay close to the data without interpretation (Sandelowski, 2000).

### 2.1 Data collection

Using convenience and snowball sampling, American nurses who worked during the COVID-19 pandemic were recruited from September 2020 – February 2022. Inclusion criteria were: Healthcare providers and/or individuals 1) who work in a healthcare setting, 2) who lived within the US, and 3) who worked during the COVID-19 pandemic. No sociodemographic exclusions were required. Participants were recruited via Social Media, specifically Facebook groups (with administrator/moderator approval), LinkedIn, and Twitter, as well as both national and state ListServs using an Institutional Review Board (IRB)-approved email.

Originally, participants who were interested in participating called the phone number included on the IRB-approved social media post or email. After calling in, participants listened to the consent form being read by one of the investigators. By pressing option 1, the participant signaled they consented to participation. Alternatively, by pressing option 2, the participant did not consent and the call ended. Lastly, by pressing option 3, the participant contacted the study team with further questions and leave a voicemail to be contacted. After consent was received, the participant was presented with three close-ended screening questions that required numeric responses that all had specific prompts: 1. What is your profession in healthcare? 2. What is your work setting? And, 3. What is your 5-digit work zip code? The participant's zip code was collected to ensure that the participant was from the US, and therefore met the inclusion criteria. Only participants who had answered all the questions with the correct prompts and met the screening criteria were allowed to leave a voicemail. Zip code demographics were not included in the final analysis to protect participant confidentiality. After consenting and meeting inclusion criteria, participants were asked to respond to the question: “Tell us about your experiences working during the COVID-19 Pandemic.” After completion of the voicemail, which was when the participant hung up the call, the voicemail was collected to a secure data platform Twilio (2022). Participants could leave voicemails up to 45 min in length, multiple times (i.e., leave voicemails detailing different experiences), and had to go through the informed consent and screening process each time.

An email option was added for participants part-way through the study to enhance recruitment after the study team began receiving email responses to recruitment posts. Instead of listening to the consent and responding to screening questions, the email participants had to read and write that they consented to their participation in the study and answer the screening questions before their email response to the prompt was recorded. No incentives were provided for participation in the study. This study was IRB-approved at the X University before recruitment.

The intent of the data collection method was to allow nurses to share their experiences at their convenience without having to set up a time for an interview. Nurses were able to freely describe their experiences, which may have lessened socially desirable responses to an interviewer (Bergen and Labonté, 2020). In addition, nurses would be able to share experiences in real-time, reducing memory recall bias. Prompted by the participants, the email option was added, since some participants preferred to share their experiences in writing.

### 2.2 Data analysis

Initial analysis included auditing the voicemails with all members of the research team. Voicemails were then transcribed using the professional service Focus Forward Transcription (2022) for a total of 238 min. After transcriptions were completed, all documents were de-identified and uploaded into the qualitative analysis software Dedoose (2022) which is a cloud-based qualitative data management and analysis platform. Transcripts were then audited independently by two investigators for any concerns related to length, completeness, duplications, and/or missing information. Content analysis was followed for all transcripts that were usable for the analysis per Elo & Kyngas (2008). Two investigators separately coded the transcripts both deductively and inductively, categorized similar codes, separated codes into subcategories, and identified overarching themes. The two investigators reviewed all codes, subcategories, and overarching themes together to reach a consensus. See Table 1 for the code tree. Saturation was achieved early in the data collection phase, but due to the ongoing nature of the COVID-19 pandemic, data collection continued to enable participants to share their experiences in case any new challenges surfaced (Corbin and Strauss, 2008).

**Table 1:** Code tree with code frequencies.

Level 1 main codes	Level 2 sub-codes	Level 3 sub-codes
Anger (69)		
At a loss (82)		
*Changing policies and procedures (86)*		
Compensation (41)		
Death (75)		
External Environment (92)		
Family Impact (66)		
Fear (72)		
*Grateful (25)*		
Guilt (13)		
Job Status (207)	Job Status Change (96)	
	Leaving the Profession (54)	
	*Overtime (28)*	
	Telehealth (30)	
	*Volunteering (13)*	
Lack of Leadership (74)		
Lack of Resources (75)		
Moral Dilemma (104)		
Negative Consequences (336)	Negative Consequences for Patients and Families (134)	*Isolation (31)*
		*Suboptimal Care Delivery (47)*
	Negative Consequences for Provider (287)	*Anxiety (63)*
		*Burnout (103)*
		*COVID-19 Infection (55)*
Nursing Staffing (89)		
Patient Nurse Interaction (131)		
Positive Outcomes of Pandemic (69)		
*Previous Epidemic Experience (8)*		
Pride (52)		
Support (154)	Negative Support (111)	
	Positive Support (41)	
Toning Down the Severity of the Pandemic (36)		
Transitions During Pandemic (137)		
Vaccines (110)		

NOTE: codes in italics are inductive codes, numbers indicate the frequency in which the code appeared within the data set.

### 2.3 Trustworthiness

Trustworthiness as outlined by Lincoln and Guba (1985) was adhered to as described below. Credibility was established through peer debriefing and multiple iterations of analysis. Transferability was addressed through the accurate description of the participant's information that they shared about themselves, their locations, and their experiences. Dependability was established through a rigorous content analysis process, and confirmability was addressed through a thorough audit trail. Reflexivity was also maintained through peer debriefing where co-investigators reflected on their positionality concering the participants and the data being analyzed.

## 3 Results

### 3.1 Socio-demographics

A total of 103 phone calls were received. Of the 103 phone calls, 83 voicemails were recorded and only 70 were included for the initial data analysis. Thirteen were removed due to being too short in length, being inaudible, missing information, or duplicates (e.g., a participant called back twice because they were cut off). In addition to the voicemails, 16 emails were also received. Five participants were removed from the final analysis as they were not nurses (e.g., certified nursing assistants), resulting in a final sample size of 81. Voicemail times ranged from 10 s – 15 min and 45 s. Table 2 includes the sociodemographic details.

**Table 2:** Sociodemographic for participants.

Variable	N(%)
Professional Role Registered Nurse or Licensed Practical Nurse Advanced Practice Registered Nurse Missing Total	67(83%) 13(16%) 1(1%) 81(100%)
Work Setting Inpatient/Hospital Outpatient/Ambulatory Long-Term Care Public Health or Community Setting Prison or Military Home Hospice Other Missing Total	37(46%) 23(28%) 8(10%) 9 (11%) 2(2.5%) 0(0%) 2(2.5%) 0 (0%) 81(100%)

### 3.2 Main themes

**Fig. 1:**
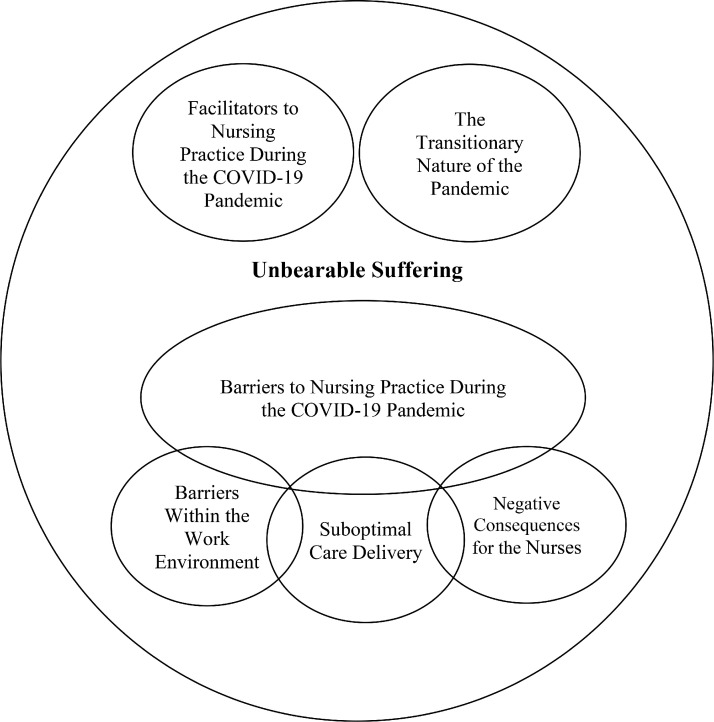
Main themes.

The overarching theme identified during the data analysis was *Unbearable Suffering*. Three additional themes were identified as illustrated in Fig. 1. While there were facilitators for nursing practice, the barriers were more common at a rate of 10–1. In the following sections, each theme will be described with illustrative quotes. See Fig. 1 for the main themes.

### 3.3 Unbearable suffering

This overarching theme of Unbearable Suffering was identified as the nurses’ experiences of witnessing the suffering of others while suffering themselves. Nurses regularly care for patients who are very acutely or terminally ill. However, the COVID-19 pandemic brought on unpreceded levels of high acuity and terminally-ill patients and death. The nurses described feeling at a loss when terminally-ill patients with COVID-19 were isolated in their hospital rooms without their families. The nurses often became the patients’ only contact, and many nurses described using their own mobile devices to call patients’ family members. The feeling of being at a loss was also due to witnessing dying patients at a rate that most participants had never experienced before. This took a toll on the nurse's mental health, and many described feeling overwhelmed, sad, and fatigued.

“Our patients stayed with us for a very long time. We do everything we can to save them but for the most part we know, most of our patients once they're intubated will die. […] We try our best to make sure people don't. It's hard. It's hard on our mental health to watch somebody say their last goodbyes. It's hard to be the person holding the iPad as the family cries and the patient cries. As they say goodbye. Many times, for the last time. It's hard to be the person who's their only point of contact. It's hard to be the family's guidance through this whole ordeal. When they have no way to actually come and see, touch and understand what's going on. It's hard to hear, well I trust in God. It's hard to hear, well I trust in something other than what we are telling people. If we could save somebody, we would. Many times, we just can't. This COVID has ripped us apart. It's ripped us apart.” (Participant #60)

While the nurses were witnessing the patients’ and families’ suffering, the nurses were suffering themselves. Many participants were fearful of contracting COVID-19, and they were afraid of infecting their families and friends. For this reason, many participants described keeping themselves in isolation when they were not working. However, many participants also described how friends and family members distanced themselves from them due to fear of contracting COVID-19. This meant that the nurses were away from their usual support system, which further exacerbated feelings of loneliness, sadness, and burnout. Moreover, most participants experienced family and friends getting COVID-19, and many experienced family, friends, and coworkers passing away from the disease.

“An additional stressor is related to family.  I have parents in their 90s in assisted living two hours away from me. There have been several hospitalizations, two rehabilitation episodes after serious injuries related to falling and frequent quarantine episodes.  There is always the conflict between taking time off work - there is limited coverage - vs. family obligations. Even though I strictly follow all masking and other protocols and have been vaccinated including booster - I worry that because of work exposure I am at risk of infecting my parents which at their age could be catastrophic.” (Participant #96)

“We're made to feel guilty for using the cleaning wipes to clean up after each patient rather than sanitizing as we should [due to lack of resources]. […] After years of being told the proper way of wearing PPE all of a sudden now we're told that we're supposed to follow their new guidelines without any really research backing that safety of the new guidelines up. And I think that created anxiety and felt like we weren't protecting again or protecting ourselves and ultimately not protecting our family. And due to that I was unable to have childcare because we didn't really feel that I was being protected at work so therefore I was exposed to the virus. And therefore, my elderly parents who usually babysat my kids were unable to do so. So I was left without help at a time that my kids were not allowed to go to school. And I had really no resources to pull from because the pandemic.” (Participant #46)

This collective suffering was especially difficult for the nurses since they continued to provide care for their patients while becoming burnt out themselves. Embedded in this experience, was the moral dilemma and guilt of not having provided adequate care for terminally ill patients, due to the patients’ isolation, and the nurses' lack of time and resources. Especially at the beginning of the pandemic, some nurses expressed feeling guilty about having instilled hope for recovery in some patients, when it later turned out that there was very little hope for recovery in many hospitalized COVID-19 patients. This suffering became all-consuming for most of the participants.

## 4 Barriers to nursing practice during the COVID-19 pandemic

### 4.1 Barriers within the work environment

The main barrier within the work environment was the deficiencies in organizational leadership. Many participants described how leadership was unsure about how to provide structure and organization during the pandemic. Subsequently, nurses experienced a significant lack of support from leadership, such as their managers, who either were unavailable or left their positions. There was often a lack of resources, such as N95 masks and gowns, and participants described how they reused and shared masks with others. Policies often changed based on these lacking resources, and it was difficult to keep up with the changing policies.

A significant challenge in the work environment was the limited nursing staffing. Many nurses left their full-time positions to pursue travel positions, and others chose to retire or work outside of healthcare. This led to mandated overtime, and while this at first was received as an opportunity to increase their income, the additional shifts soon began to wear the nurses out. They did not feel supported by their leadership and they did not feel adequately compensated for the extended working hours in a hazardous work environment. Moreover, travel nurses filled the open positions which often created tension, especially on hospital units. While travel nurses cared for their patients, they were unfamiliar with the culture and job responsibilities unique to each setting, and these responsibilities fell back on the full-time nurses. To that end included the frustrations among full-time nurses that travel nurses earned higher salaries, some reporting as much as three times their wage. Nurses in full-time positions described being overwhelmed with responsibilities due to working with travel nurses, working overtime, and a lack of unit management, which led to frustration and anger.

“Not being compensated, not feeling there's anyone advocating for the well-being of the nurses; just constantly being asked to do more, and more, and more; and take on more roles; and care for sicker patients; and expanded ratios; and having paired devices and tripled assignments. In no other profession would people be asked to do what we're being asked to do. And I used to think that nursing was my calling. And here I am three years into my profession, and I am really thinking about doing something completely different, because I cannot have my altruism taken advantage of like this anymore. I feel like I have more respect for myself now, and I just don't want to be treated like such garbage by a system that's so broken. So really trying to figure out how to get out, which is heart-breaking because I used to love taking care of patients. And now, I just feel like I am complicit and doing something evil by torturing these people who are never going to survive their hospitalization.” (Participant #50)

Another component embedded in this theme was how the severity of the pandemic was toned down by others. These included co-workers, family members, patients, patients' families, and often the public in general. Experiencing how some patients and their families did not believe COVID-19 to be dangerous or even existed, making it very difficult for the participants to practice, and feel comfortable outside of their work environments. They described being caught in the battle between believers and non-believers of the pandemic and being ridiculed from both perspectives. Many participants described having cared for dying parents of young children, where the parents had refused to get vaccinated or receive recommended medical treatment due to these beliefs and then passed away after realizing that COVID-19 could be fatal. This battle between believers and non-believers often occurred among co-workers as well, especially when vaccine requirements were put into place. Further, this battle continued in the public, where they never knew if they would be praised for their work during this time or ridiculed for being part of a conspiracy. Being unintentionally caught in the middle of this battle made it difficult for the nurses to both provide adequate care to their patients and difficult for them to recover and recharge after difficult shifts at work. 

“This profession has been something that many of us have worked so hard for. Many of us are now considering leaving because as the pandemic went on and vaccines became available. Where we only had other preventative measures in place, people just decided not to do it. It's those people that continue to keep coming in. Those people who continue to keep denying and it breaks our heart every time. I've seen so many people who should have not died young. Die young. I've seen so many families lose beloved family members way too early in their lives. There are people who I've seen die that will haunt me the rest of my life.” (Participant #60)

Many participants shared their deep frustrations in the voicemails, and let their anger come through in their firm, loud voices:

“Some of my co-workers refused the vaccines for the familiar wrong reasons.  Eventually several of them quit, leaving those of us who believe in science and believe keeping our patients safe from catching a virus that could kill them to work with fewer staff members, heavier loads, and lots of exhaustion.  In July, like 17 other staff members on my unit, I got COVID, was symptomatic, lost 10 pounds in a week.  I had been vaccinated in January.  When I returned to work absolutely nothing had changed.  Patients to my face continued to tell me that the vaccines didn't work, that they would never take the vaccines (but they would smoke cigarettes, drink alcohol, drink Mountain Dew, eat a terrible diet, take tons of over the counter drugs, etc.). And so it went.” (Email response 11.12.2021)

### 4.2 Suboptimal care delivery

The participants continuously described how the patients received suboptimal care. In the outpatient settings, participants described how they began seeing patients via telehealth, which was a difficult transition for both providers and patients. Patients being seen for mental health illnesses missed their appointments due to a lack of access to the required technology. In inpatient settings, suboptimal care involved poor coordination of care, prolonged wait times for testing and procedures, and a lack of resources and equipment, such as PPE. What the participants perceived as the worst consequence of the pandemic for the patients, was isolation. With no visitors being allowed in many healthcare settings, patients became deeply isolated in their illness experiences. This was described in a variety of settings, including hospital units, nursing homes, and outpatient settings. The nurses became their patients’ families during this time. Many patients, especially at the beginning of the pandemic, did not have online video conferencing, such as FaceTime, available to them. This loneliness experienced by the patients was especially difficult for the nurses, which made them feel helpless and hopeless, which further added to their own suffering. When participants shared these experiences, they were often crying as they were leaving their voice messages, and moments of silence were common, where the participants regained their ability to speak.

“Coding patients was common. It also became predictable which patients would die. This increased our determination to help the patients but was also disheartening when they did pass. The intubated COVID patient were very sick patients to care for typically needing multiple IV drips, including sedation, paralytic and pressure meds. Many needed dialysis, which was a strong indicator of a terminal state in our battle to save the patients. Of the 13 we had on a vent during the Delta surge, 9 passed away. This was the most challenging period of the pandemic for me due to the devastation the COVID virus wracked on patients. Patient did not respond to our touch or speech, due to the paralytics. For me severe COVID infections were a very dehumanizing process. Patient could not have family at any point in their hospitalization, they were isolated from everyone but direct care staff who were in full PPE; and once intubated they no longer had control of any basic human functions. The 9 patients that died, never woke up or interacted with the world again once they were intubated.”  (Email response 12.21.2021)

### 4.3 Negative consequences for the nurses

The consequences of the suboptimal work environment and the barriers to providing optimal nursing care had significant negative consequences for the nurses. This included feeling constantly either frustrated, angry or at a loss. Due to working many hours and shifts a week, the nurses were not able to process or recover from these feelings before their next shift. To that end, many participants had changed their jobs, seeking less stressful nursing jobs in other settings, and many participants described the desire to leave the nursing profession. For example, in describing their experience working during the COVID-19 pandemic, and their negative feelings toward being a nurse:

“I think what makes me so frustrated is that I love being a nurse. And I hate it now. I hate it so much. And I take so much pride in how I interact with people, and how I treat people, and how I care for people, and I don't have that anymore. I can't- I have a very limited amount of empathy and limited amount of resources that I can extend to other people because I need it for myself.” (Participant #49)

More severe consequences were revealed among some nurses in the study. Some nurses shared how they coped with the stress from work by overusing alcohol, necessitating external support resources:

“It has caused me to do some things I [never] thought I would do […]. I've always considered myself a resilient enough individual to be able to deal with things without going to counseling. I have been to counseling. I have joined AA. I don't think I had a drinking problem prior to this, but I find the support that you get from Alcoholics Anonymous to be very wonderful overall not just for drinking but just as far as being a human being. It's been a wonderful experience that's come out of a very horrible experience for me.” (Participant #70)

Some participants shared experiences of suicidality that they claimed were directly related to their experiences working as a nurse during the COVID-19 pandemic.

“During spring 2021, I became so disparaged over coming into work, feeling like I was doing nothing to help my patients, struggling to listen to coworkers push non-scientific talking points about the vaccine, and leaving feeling burnt out that I began planning to end my life. I was depressed all the time and could barely get it together to shower and make it to work. I quit in September 2021 with no other job in place and no other source of income because my partner was concerned I was going to commit suicide. I felt used, abused, and traumatized. I'm slowly processing it, but I'm already at the point where I'm looking into other professions down the line. When people ask me if they should become a nurse I say no.” (Participant #60)

The barriers within the work environment, the consequences of the work environment, and the public external environment became a vicious circle. The barriers became continuously more overwhelming, leading to burnout, and even the severe consequences of alcohol abuse and suicidality. The participants struggled to cope with these experiences, and most participants shared their thoughts about retiring early or leaving the nursing profession and healthcare in general.

### 4.4 Facilitators to nursing practice during the COVID-19 pandemic

While the vast majority of the experiences shared by the participants in this study were negative, many nurses shared positive experiences as well. Many nurses experienced their coworkers, unit managers, and other staff working together to tackle this new situation. They described improvements in teamwork, both among nurses and in interdisciplinary teamwork. They felt proud of how their organization and their coworkers overcame this difficult situation, and these positive experiences in turn provided energy to keep going during these difficult times. Some nurses felt supported by their coworkers and leadership, which left them with feelings of gratitude. Nurses with years of experience described how they were reminded why they became a nurse, and some newly graduated nurses described being proud of having chosen the right profession and were grateful for the opportunity to learn in such a unique situation. These positive experiences also included descriptions of how the nurses felt supported and appreciated by patients and families in addition to their own families and friends. In addition to their regular work, some participants volunteered in local vaccine clinics, which promoted feelings of pride. Many nurses felt hopeful and grateful when the COVID-19 vaccines were launched.

“I think COVID was hard. But it reminded me of why I wanted to be a nurse […]I'm almost ready to retire. I'm glad I got to nurse during COVID. I just really wanted to know that I could do it, and I felt I had something to offer […]. I actually look back on it a little bit fondly, and I'm so glad I had the experience. I wouldn't have wanted to miss out.” (Participant #67)

A few participants described previous experiences with epidemics, such as flu and Ebola. They used these previous experiences in organizing and overcoming their current pandemic work. While the COVID-19 pandemic was an unprecedented situation, some nurses felt somewhat prepared for the situation, and they were able to use their previous experiences to maintain a better work environment for themselves, their co-workers, and their patients.

### 4.5 The transitionary nature of the pandemic

Most of the participants described how their work tasks had changed throughout the pandemic. This included makeshift isolation procedures in hospitals and nursing homes, and changing from in-person visits to telehealth in outpatient settings. Some participants shared how hospitals at the beginning of the pandemic were well prepared and overstaffed with limited patient loads, yet a few months later hospitals were at patient capacity with a nursing shortage.

“At the beginning of the outbreak of the pandemic, everybody was really nervous. We overprepared, shut down surgeries, uped staff, hired a bunch of travelers. Within the first three months, we found nothing, no positive COVID or overstaffed every day. And slowly but surely, we started elective surgeries and started to see more and more COVID patients come in […]. By then, we were already working short-staffed and getting hit pretty hard. Over the last two months, we have been a nonstop hit by COVID patients, have been understaffed. My nurse co-worker friends have gone out on stress leave, being overwhelmed by the processes. We have turned regular rooms into makeshift isolation rooms with plastic, zipper doors, and HEPA filters. It is very overwhelming and exhausting.” (Participant #45)

With the data collection spanning over 18 months, the participants’ experiences changed throughout this time, especially regarding the COVID-19 vaccines. Participants during the first nine months of the pandemic were hopeful about the vaccines: *“I have had my vaccine, so that will hopefully, if everybody will get that, [COVID-19] will decrease.”* (Participant #34). During the second year of the pandemic after the roll-out of the vaccines, the nurses became more frustrated about how many individuals, both nurses, patients, and the public, refused the vaccines; *“I got vaccinated as soon as I could in January and watched in dismay as about half of my unit never got vaccinated due to varying reasons, but none of them medical.”* (Email response 01.12.22).

The nurses participating in this study were from all over the US. Over 18 months of data collection, the participants’ experiences reflected the ebb and flow of the COVID-19 pandemic in different states at different times. Participants would call in from one state and share the overwhelming experiences of the peek of the pandemic in their state. At the same time, participants in other states shared experiences of how the numbers of COVID-19 patients were decreasing and participants were relieved about how workflows were getting back to normal levels. Broadly sharing the similarities and differences that nurses experienced in real-time across the US during COVID-19 helps understand the impact on the nursing profession and where to focus future efforts.

## 5 Discussion

The primary finding from this study was that nurses were witnessing and experiencing unprecedented suffering that became unbearable while working during the COVID-19 pandemic. This finding is supported by current literature on nurses' experiences working globally during the COVID-19 pandemic (Catania et al., 2021;Galanis et al., 2021;Halcomb et al., 2020;Stravropoulou et al., 2022). Nurses in this study described not just physical human suffering but also bore witness to patients and their loved ones' psychological suffering related to mandated isolation. This same phenomenon was also described by Bethel et al. (2022) where critical care nurses described witnessing unparalleled patient deaths. Similar to extant research, the nurses in our study experienced fear of the unknown related to COVID-19 (Bethel et al., 2022;Liang et al., 2021;Littzen, 2021), impacting the nurses' mental health fatigue. Moreover, the nurses described significant impacts on their work-related well-being. Littzen (2021), described the negative consequences of morally difficult decision-making during the pandemic as having lasting impacts on their well-being. Similarly, Fitzpatrick et al. (2022), described a significant association between moral injury and the well-being of nurses’ working during the COVID-19 pandemic, meaning that, as moral injury increased, nurses’ well-being decreased. Laher et al. (2022) discovered that while moral injury was present before the pandemic, the impact of the pandemic likely exacerbated its prevalence. In this study, the described unbearable suffering the nurses experienced may demonstrate a beginning understanding of the prevalence of the moral impacts of nursing during the COVID-19 pandemic.

While the experiences reported in this study, including the unbearable suffering of both nurses, patients, and families, and the barriers in the work environment, are similar to those reported in other studies (Fernandez et al., 2020;Fernandez-Basanta et al., 2022;Zipf et al., 2021), the participants in our study disclosed more extreme experiences than participants in other studies may have. Burnout, fatigue, and moral distress among nurses have been previously reported, but participants in our study were very transparent in sharing how their experiences led to the overuse of alcohol, receiving mental health therapy and treatment, and even suicide ideation. In a similar study, Maben et al. (2022) described that the nurses they interviewed had higher rates of alcohol consumption, but did not discuss alcohol abuse or the need for external support resources. The participants in our study may have felt more comfortable reporting deeply personal experiences due to the anonymous nature of the data collection by leaving a telephone message instead of being interviewed. Our data collection method may have decreased socially desirable responses (Bergen, and Labonté, 2020), and more honest experiences were shared. The prevalence of extremely negative outcomes among nurses working during the COVID-19 pandemic may not have been captured in previous research, yet these outcomes warrant immediate attention in both research, policy, and practice.

Another finding from our study was the transitionary nature of the pandemic. Specifically, we found that while nurses may have had similar experiences working during the COVID-19 pandemic, these experiences occurred at different points in time due to their geographical location. In public health, the geographical implications of COVID-19 have been widely reported by both state and federal organizations (Centers for Disease Control and Prevention, 2023). In nursing, concerns of nurses related to forthcoming changes due to the COVID-19 pandemic have been reported (e.g., fear of rapid and unpredictable changes) (Jun and Rosemberg, 2021;Kelly et al., 2022; Fernandez-Basanta et al., 2022), but the transitionary nature of the pandemic has not been described. This finding is especially important as it highlights the need for proactive interventions for future crises (e.g., pandemics) to support nurses, such as incidence reporting, the fair distribution of resources, and mental health support services.

Facilitators to nursing practice were described in this study as teamwork among all healthcare providers, feeling supported by co-workers, leadership, patients, and families, having a sense of pride and feelings of gratitude, and being hopeful. Being positively and morally supported is important for nurses’ well-being (Littzen, 2021; Rainbow et al., 2021). Feeling supported in the work environment influenced nurses' intention to stay in the workforce during COVID-19 (Sanner-Stiehr et al., 2022). Furthermore, resilience, a process of recovering from burnout or stress (Rink et al., 2022), was reported to be at a moderate level during the COVID-19 pandemic (Baskin and Bartlett, 2021). Contributors associated with resilience included work engagement and social support that positively impacted post-traumatic stress disorder, anxiety, and depression (Baskin and Bartlett, 2021). Nurse resiliency models integrated into the organizational structure of healthcare systems at the individual, organizational, and community levels are a way to protect nurses’ well-being (National Academies of Sciences, Engineering, and Medicine, 2021). In addition to institutional support, having a dedicated resource monitoring plan and employee support plan across institutions contributes to a safe and calm environment (Sanner-Stiehr et al., 2022).

Nurses in this study reported feelings of gratitude, feelings of being thankful, and hope. Gratitude practices have been shown to help with nurses’ mental health (Melnyk et al., 2020). In our study, some nurses were reminded of why they wanted to be a nurse. Similar findings have been found globally in frontline nurses’ caring experience in COVID-19 units, specifically in South Korea (Shin and Yoo, 2022). As previously discussed, maintaining and facilitating the health and well-being of healthcare workers is important to maintain the nursing workforce, but also improves safety, the care of patients, and healthcare outcomes (Sexton et al., 2021;Rehder et al., 2021).

Lastly, while this study was conducted among nurses in the United States, similar research has been conducted among nurses around the world (Bergman et al., 2021;Catania et al., 2021;Halcomb et al., 2020). The trend is clear; nurses worldwide are experiencing unbearable suffering, and as a result, are facing new-onset anxiety, stress, fatigue, and significant burnout - placing them at risk for more severe consequences such as alcohol abuse and suicide (Bethel et al., 2022;Catania et al., 2021;Hur et al., 2022;International Council of Nurses, 2021;Littzen, 2021). Addressing this problem now is imperative since the long-lasting effects of nurses' suboptimal well-being on system or patient outcomes, global health, as well as the longevity of our discipline, are unknown.

### 5.1 Strengths and limitations

The strengths of this study included the innovation of methods including multiple modes of recruitment (both written and oral) that had no time restriction. Additionally, the form of data collection enabled participants to share their experiences in a cathartic way which was a form of debriefing. Lastly, participants were able to contribute to research asynchronously without sacrificing qualitative trustworthiness, thus being participant-centered and rigorous.

The limitations of this study include the lack of context or follow-up enabled by the one-sided nature of the recruitment method. Additionally, we did not calculate the average time of the voicemails, as the variability in length of time would have resulted in an unrepresentative average. There is also the potential for self-selection bias for those who did participate. Moreover, those that did not participate may have had a different experience working during the pandemic and may be at risk for more negative consequences due to cognitive load. Some telephone messages were very short (<1 min), which threatens the depth of data. While social desirability bias cannot be ruled out for participants sharing their stories knowing the context of the research, the participants in this study shared more severe and extreme experiences than those reported in other studies. This form of data collection indicates an ability to capture more intense experiences than other methods of data collection.

## 6 Conclusions

The primary finding of this study was that nurses experienced and witnessed unbearable suffering while working during the COVID-19 pandemic, as well as its transitionary nature. Furthermore, some of the nurses indicated that they may leave the profession of nursing based on their experiences. Future research should consider the long-term impacts of this unbearable suffering on nurses. Intervention research should be considered to support nurses who have worked during the COVID-19 pandemic and mitigate the potential long-term effects.

## Funding sources

No external funding

## Declaration of Competing Interest

The authors declare that they have no known competing financial interests or personal relationships that could have appeared to influence the work reported in this paper.
